# Addicted, attached, or just delegating? A scoping review on “problematic artificial intelligence use”

**DOI:** 10.3389/fpsyg.2026.1900953

**Published:** 2026-07-09

**Authors:** Francesca Maria Dagnino, Chiara Fante, Vittorio Guerrieri, Marcello Passarelli

**Affiliations:** 1 Institute for Educational Technology, National Research Council of Italy, Genoa, Italy; 2 Italian Institute of Technology, Genoa, Italy

**Keywords:** AI addiction, AI dependence, emotional attachment, generative AI, large language models (LLMs), overreliance, problematic AI use, scoping review

## Abstract

**Background:**

The rapid diffusion of generative and conversational AI has raised concerns about problematic AI use and AI dependence. This led to a proliferation of studies addressing the problem, despite the lack of a common framework. This scoping review maps: (RQ1) definitions, (RQ2) measurement approaches, (RQ3) correlates and outcomes, and (RQ4) preliminary evidence across operationalization.

**Methods:**

Following PRISMA-ScR guidelines, we searched Web of Science and Scopus using predefined strings on artificial intelligence and problematic use. Thirty-seven empirical peer-reviewed studies were included.

**Results:**

Findings highlight inconsistent terminology and considerable heterogeneity in how problematic AI use is conceptualized, exacerbated by a frequent gap between constructs and operationalization that limits interpretation of outcomes. After recoding measures by their substantive operationalization, we analyzed evidence for three main strands: (1) behavioral addiction and/or compulsive use, consistently associated with depression, loneliness, social anxiety, escapism, flow state and low self-esteem/self-efficacy, where younger age and male gender emerge as risk factors; (2) cognitive (over)reliance, linked to performance expectations, academic stress/frustration of needs and literacy/trust in AI, with converging evidence of an erosion of downstream skills (and a decline in performance when AI is unavailable); and (3) Psychological and emotional dependence, associated with loneliness, anxious attachment, anthropomorphizing, and the perception of warmth/emotional intelligence, reliability and availability of AI.

**Conclusion:**

The field is fragmented and would benefit from clearer construct specification, AI-specific validated scales capturing all features of the phenomenon, and more longitudinal and experimental designs to clarify causal mechanisms and support safer system design.

**Systematic review registration:**

OSF, accessible at: https://osf.io/cxqrz

## 1 Introduction

In recent years, the artificial intelligence (AI) sector has experienced substantial expansion; technology companies have been developing and releasing AI models at an accelerating rate, while citizens are increasingly encouraged to access and use these systems through their personal devices (Maslej et al., 2025). This rapid, large-scale diffusion is enabling people with different backgrounds and levels of awareness to use advanced systems that can be queried using natural language. These systems can generate responses, propose solutions, and support decision-making processes within seconds, a possibility that undoubtedly represents a valuable resource in everyday life. Although these features offer clear benefits in professional and educational activities, they also introduce emerging risks related to overreliance on algorithmic systems, fostering a tendency toward excessive dependence on such technologies (Passi and Vorvoreanu, 2022; Zhai et al., 2024). This risk is compounded by the capacity of the AI to simulate empathy, attention and emotional engagement. These capabilities heighten the risk of illusory reciprocity and anthropomorphizing (Placani, 2024), thereby blurring the boundaries between human actors and algorithmic systems. The relational dimension of problematic AI use actually predates the current wave of large language models. Research on early “social chatbots,” such as Replika (launched in 2017, and nowadays running on LLM technology), had already demonstrated that users can develop strong emotional attachments and parasocial relationships to systems explicitly designed for companionship (Skjuve et al., 2021; Pentina et al., 2023), with potential risks including addiction and detrimental effects on real-life relationships (Xie and Pentina, 2022; Laestadius et al., 2024). A key design feature of these systems was their tendency to provide unconditional emotional validation and non-judgmental feedback, a pattern now referred to, in the context of LLMs, as “AI sycophancy”: the tendency of the model to mirror user opinions, reinforce biases, and display excessive agreeableness (Cheng et al., 2026; Wei et al., 2024). While anthropomorphizing leads users to attribute human qualities, such as empathy, to the machine (Placani, 2024), sycophantic behavior further amplifies this effect by creating a “perfect” conversational partner that rarely challenges the user. The frictionless nature of AI-mediated interaction renders the complexities and demands of authentic human relationships increasingly unappealing by comparison, potentially contributing to social withdrawal and phenomena sometimes described as “technological folie à deux” (Dohnány et al., 2026; Sharma et al., 2024).

In the current wave of generative AI models, which is leading to widespread diffusion for educational and professional practices, users who initially adopt AI for instrumental purposes may gradually develop emotional engagement patterns without explicit awareness (Zhang Y. et al., 2025). Moreover, the very features that foster emotional attachment, constant availability, non-judgmental responsiveness, and frictionless interaction, together with the perceived potential and ability of AI to perform tasks efficiently, also facilitate a subtler form of problematic AI use: the habitual delegation of core cognitive operations to the system, as a maladaptive form of “cognitive offloading” (Risko and Gilbert, 2016). In educational settings, emerging evidence suggests that such delegation may produce a dissociation between task performance and actual learning: students using AI tools answer more problems correctly while demonstrating poorer conceptual understanding (Bastani et al., 2025), a pattern consistent with skill atrophy concerns raised in the broader literature on AI overreliance (Abbas et al., 2024; Niloy et al., 2024).

In this evolving landscape that includes cognitive and affective reliance on AI, the concept of “problematic AI use” is becoming increasingly relevant. However, despite its frequent appearance in both public and academic debate, the term still lacks a shared and consistent definition, as existing analyses tend to focus on specific and partially overlapping conceptualizations of problematic AI use, such as excessive reliance, addiction or addiction-like patterns (Al-Obaydi and Pikhart, 2026; Passi and Vorvoreanu, 2022; Zhai et al., 2024). Furthermore, some of the terms mentioned above—sycophancy, cognitive offloading, parasocial relationships, anthropomorphism—come from different disciplinary traditions and, sometimes, imply different conceptualisations on human-AI interaction and problematic AI use. Rather than representing a limitation, this conceptual vagueness reflects the heterogeneity of the manifestations that can be grouped under the label of problematic AI use, which are often analytically distinct but empirically interconnected. Beyond this conceptual ambiguity, a further debate concerns whether problematic AI use should be understood as a distinct phenomenon or as a manifestation of problematic technology use, similarly to social media addiction, excessive smartphone use, or compulsive Internet-related behaviors (Kooli et al., 2025).

Some researchers suggest that existing frameworks adequately capture the maladaptive dynamics that emerge around AI (Huang et al., 2024), while others argue that the uniqueness of generative and conversational systems, such as their adaptive feedback and anthropomorphic cues, may lead to qualitatively different patterns of engagement (Mahari and Pataranutaporn, 2025; Shen et al., 2026). Beyond these theoretical disagreements, in addition to conceptual issues related to the nature of problematic AI use, an emerging body of research is exploring the correlates of maladaptive patterns of interaction with AI, with the intent of identifying potential risk factors. Mapping which variables are receiving systematic attention and whether consistent patterns are emerging across different lines of research could be valuable to highlight gaps in the research. Furthermore, assessing whether differences in definitions and measurements stem from different theoretical conceptualizations could provide insights into the actual level of fragmentation of this emerging field.

Taken together, the conceptual and methodological uncertainties described above underscore the need for a structured synthesis of the emerging literature on problematic AI use. The multidimensional nature of the phenomenon, coupled with the tendency of existing studies to focus on specific features of this phenomenon, makes it difficult to clarify how problematic AI use is currently defined, conceptualized, and operationalized, as well as to determine which individual, social, and contextual factors have been studied as correlates. To date and to the best of our knowledge, no comprehensive review has systematically addressed these issues, as they only consider narrower definitions of problematic AI use (Al-Obaydi and Pikhart, 2026; Passi and Vorvoreanu, 2022; Zhai et al., 2024). This scoping review includes manuscripts that describe the use as ‘excessive’ or ‘maladaptive’ and report associated negative cognitive, emotional, or social consequences. Consequently, it aims to answer the following research questions (RQs):

1. How is problematic AI use defined and conceptualized in the literature?
2. How is problematic AI use measured?
3. Which correlates and associated outcomes have been investigated for each different *definition* of problematic AI use?
4. What preliminary evidence is available on correlates and associated outcomes for each *operationalization* of problematic AI use?

It was considered that a scoping review represented the most appropriate design, given the heterogeneity of theoretical perspectives and the broad concept of ‘problematic AI use’, factors which, taken together, preclude a comprehensive meta-analytical analysis (Arksey and O’Malley, 2005; Tricco et al., 2018). The gaps identified in the evidence base are therefore highlighted as priorities for future research.

Notably, our study aims to operate at both the level of definitions (RQs 1 and 3) and of operationalizations (RQs 2 and 4), clearly distinguishing between the two, as the complexity and emerging nature of the field leads, as will be argued in the following Sections, to a mismatch between these two levels.

## 2 Methods

The search was carried out following the indications of the Preferred Reporting Items for Systematic Reviews and meta-analysis extensions for Scoping Reviews (PRISMA-ScR) (Tricco et al., 2018). A review protocol was built by the researchers and pre-registered on the Open Science Framework (OSF), accessible at https://osf.io/cxqrz/overview?view_only=22aa909630f6448297410ad788692ea2.

### 2.1 Search strategy

Two electronic databases, Web of Science (WoS) and Scopus, were searched using predefined search strings. The same keywords were used across databases, with search strings adapted to the specific syntactic requirements of each platform. The search strategy encompassed two main conceptual domains: artificial intelligence and problematic use. Searches were performed considering titles, abstracts, and keywords. Since we wanted to approach the issue from a multidisciplinary perspective, the two databases were selected as they are among the largest and most widely used multidisciplinary bibliographic databases (Falagas et al., 2008; Singh et al., 2021). Their combined use was considered capable of providing broad coverage of peer-reviewed scientific literature while facilitating the retrieval of relevant studies.

The string used for Scopus was the following: TITLE-ABS-KEY [(ai OR llm OR chatgpt OR “artificial intelligence” OR chatbot OR “conversational agent” OR llms OR replika) AND (addiction OR compulsive OR “problematic use” OR “excessive use” OR “behavioral addiction” OR “pathological use” OR “impaired control”)] OR TITLE-ABS-KEY (“AI depend*” OR “artificial intelligence depend*” OR “chatbot depend*” OR “llm depend*”) AND [LIMIT-TO (DOCTYPE,"ar”) OR LIMIT-TO (DOCTYPE,"cp”) OR LIMIT-TO (DOCTYPE,"re”)].

The string used for WoS was the following: TS = {[(ai OR llm OR llms OR chatgpt OR chatbot OR “artificial intelligence” OR “conversational agent” OR replika) AND (addiction OR compulsive OR “problematic use” OR “excessive use” OR “behavioral addiction” OR “pathological use” OR “impaired control”)] OR (“AI depend*” OR “artificial intelligence depend*” OR “chatbot depend*” OR “llm depend*”)} AND DT = (ARTICLE OR PROCEEDINGS PAPER OR REVIEW).

The search was carried out in July 2025 without any time and language restrictions.

Retrieved records were exported for duplicate removal and subsequent title and abstract screening. A subset of 100 papers was screened by two reviewers to convene selection criteria and check agreement. Inter-rater reliability on the independently double-screened abstracts was high (Cohen’s *κ* = 0.95; 98% agreement). Discrepancies were resolved through discussion. Afterwards, the four researchers independently screened titles and abstracts. Studies were flagged for full-text retrieval if they met the predefined inclusion criteria. Following the recommendations of Levac et al. (2010), researchers convened at the beginning, midpoint, and end of the abstract screening process to address challenges, clarify uncertainties, and, if needed, refine the search strategy. This phase resulted in the identification of potentially relevant studies, which were then divided among the researchers for independent assessment. Full-text articles were retrieved and independently evaluated for eligibility by the four researchers in accordance with the predefined inclusion and exclusion criteria. Uncertainties were resolved through discussion.

### 2.2 Inclusion criteria

Studies were included in the scoping review if they:

1. Were primary studies employing qualitative, quantitative, or mixed methods.
2. Were peer-reviewed publications in journals or conference proceedings.
3. Studied the phenomena of problematic AI use, overreliance on AI, AI addiction and/or AI dependency.
4. Explicitly studied generative AI/LLMs, or used the term “AI” without further clarification (under the rationale that the current wave of generative AI led to the general term “AI” being increasingly used for referring to generative AI specifically, except in technical fields).
5. Were published in English.

### 2.3 Exclusion criteria

Studies were excluded if they:

1. Mentioned problematic AI use without addressing it as a primary research objective (e.g., it emerged as an unexpected theme in a qualitative study).
2. Were theoretical or conceptual in nature (e.g., narrative reviews, commentaries, or framework papers) and did not report original empirical data. Note that the search query explicitly included reviews and meta-analyses, but these were retrieved to better define the theoretical background and were not systematically analyzed, unlike primary studies.
3. Were explicitly limited to non-generative AI, such as studies on dependence due to personalised recommendation systems.

### 2.4 Data charting process and data items

In line with Levac et al. (2010), data charting was conducted independently by the four reviewers using a structured form developed by the authors to systematically capture key study characteristics and variables relevant to the review objectives. Data charted from the included sources of evidence were related to:

- *General study characteristics*: title, authors, year of publication, country.
- *Methodological characteristics*: study design, data collection methods, samples.
- *AI technologies examined.*
- *Findings relevant to the review objectives*: definition of problematic AI use, focus of the study, key results and outcomes, especially correlates of problematic AI use and associated outcomes if studied.

Supplementary Table A1, reports the results of data charting.

In line with established scoping review guidance, a formal assessment of methodological quality was not undertaken, as the aim of this review was to map the existing literature rather than evaluate the strength of evidence. However, to adequately address our research questions, an in-depth analysis was conducted on the operationalization of problematic AI use, as this is an aspect closely linked to its conceptualization.

## 3 Results

### 3.1 Selection of sources of evidence

**Figure 1:**
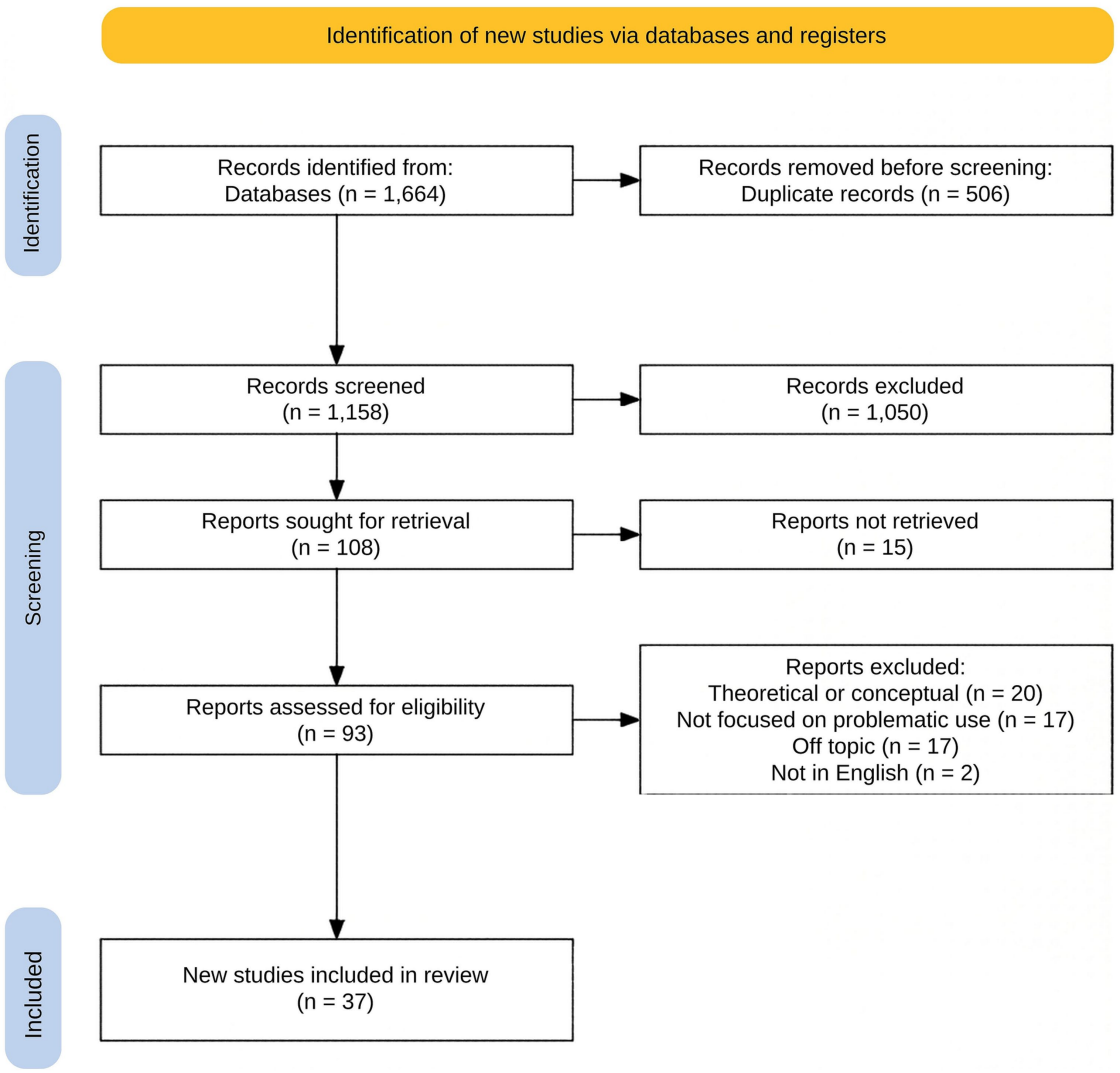
PRISMA flowchart. Produced using Haddaway et al. (2022) package and app.

Figure 1 reports the PRISMA flow chart of retrieved studies. From 1,664 initial records, the selection process led to the identification and retrieval of 37 primary studies.

Table 1 reports the studies included in the review with the abbreviation (A01-A37) used throughout the results section to reference them.

**Table 1:** Paper codes, declared focus and given definitions using other terms.

#	Authors (Year)	Declared focus	Terms used in definition	#	Authors (Year)	Declared focus	Terms used in definition
A01	Estrada-Araoz et al. (2025)	Dependenc*	Compulsive use	A20	Zhong et al. (2024)	Dependenc*	Not defined
A02	Huang et al. (2024)	Dependenc*	Addiction	A21	Hu et al. (2023)	Problematic use	
A03	Abbas et al. (2025)	Addiction		A22	Morales-García et al. (2025)	Dependenc*	Compulsive use
A04	Chen et al. (2025)	Dependenc*	Addiction	A23	Frenkenberg and Hochman (2025)	Dependenc*	Addiction
A05	Duong et al. (2024)	Compulsive use		A24	Wijaya et al. (2024)	Dependenc*	(over)reliance
A06	Yao et al. (2025)	Problematic use		A25	Xie et al. (2025)	Addiction	
A07	Long et al. (2025)	Dependenc*		A26	Buniel et al. (2025)	Dependenc*	(over)reliance
A08	Zunaidah et al. (2023)	Dependenc*, (over)reliance	Not defined	A27	Marriott and Pitardi (2024)	Addiction	
A09	Morales-García et al. (2024)	Dependenc*	(over)reliance	A28	Liao et al. (2025)	Dependenc*	
A10	Yu et al. (2024)	Problematic use	Dependence	A29	Maral et al. (2025)	Problematic use	Compulsive use
A11	Zhang et al. (2024)	Dependenc*	(over)reliance	A30	Ali et al. (2024)	Compulsive use	
A12	Tseng et al. (2025)	Dependenc*	Not defined	A31	Capinding (2024)	Dependenc*, (over)reliance	Not defined
A13	Park et al. (2025)	Dependenc*, (over)reliance	Not defined	A32	Zhang Y. et al. (2025)	Dependenc*	Reliance
A14	Petrič (2024)	Problematic use		A33	Acosta-Enriquez et al. (2025)	Dependenc*	Compulsive use
A15	Zhou and Zhang (2024)	Addiction		A34	Zhang and Xu (2025)	Dependenc*	(over)reliance
A16	Zhang X. et al. (2025)	Dependenc*	Not defined	A35	Capinding and Dumayas (2024)	Dependenc*	Not defined
A17	Zhang D. et al. (2025)	Dependenc*	(over)reliance	A36	Hu et al. (2025)	Compulsive use	Not defined
A18	Xie et al. (2023)	Dependenc*	Addiction	A37	Biswas and Murray (2024)	Reliance	
A19	Li et al. (2025)	Dependenc*	(over)reliance				

### 3.2 Study context and characteristics

As far as the year of publication is concerned, we observe an increase in the attention towards the topic: only three papers were published in 2023, while 13 in 2024 and 21 papers were published in 2025. Studies were carried out mainly in China (17 studies), followed by a small group in Perú (4 studies) and in the Philippines (3 studies); other countries are represented by one or two studies. From the methodological point of view, studies were predominantly cross-sectional, with a prevalence of quantitative approaches; almost all were based on surveys, using self-developed questionnaires or scales developed in the same or previous studies, only one used an observation rubric. With respect to the systems under study, when specified, the included corpus concerned predominantly considered general-purpose large language model assistants (e.g., ChatGPT) and LLM-based companion chatbots (e.g., Replika). Samples ranged from 34 to 3,843 subjects; more than half of the studies have been carried out with college or university students (20 out of 37 studies), while the larger sample (3843) was composed of adolescents. Preservice and in-service teachers were targeted by three studies. Findings relevant to the review objectives are presented and discussed in the following subsections.

### 3.3 RQ 1—how is problematic AI use defined and conceptualized in the literature?

In this review, we refer to multiple expressions of ‘problematic AI use’ to consider the variety of conditions discussed in the literature. Indeed, different terms were adopted in the studies to name the use of AI that begins to have a detrimental impact on cognitive, emotional, or social well-being: “reliance,” “overreliance,” “dependency,” “addiction,” “compulsive use,” are often cited. Sometimes authors provide an explicit definition of AI use using these terms; other times, they provide no explicit definition, but their conceptualization, i.e., their theoretical model of the phenomenon, can be inferred. In both cases, we observe a lack of uniformity in terminology, which results in using terms interchangeably and in the association of partially overlapping concepts (see Table 1).

Most of the authors state that they investigate AI dependence or dependency (A01; A02; A04; A07; A08; A09; A11; A12; A16; A17; A19; A20; A22; A23; A24; A26; A28; A33; A34; A35) but the provided definitions often include other words and concepts. For example, dependence and addiction are sometimes treated as synonyms (A02; A04; A18; A23); in A23, published in 2025, authors affirm that this correspondence is now generally established: “Earlier research often used terms like “overreliance,” “addiction,” and “dependency” interchangeably […] AI dependency is now being recognized as a specific form of behavioral addiction” (page 8). Conversely, other authors highlight the different meanings of the two terms, as in A19, likewise published in 2025: “Addiction often entails severe physiological and psychological impacts, potentially leading to the deterioration of individual health, social dysfunction, and even legal issues. In contrast, dependence might induce certain negative effects, such as poor time management or excessive reliance on technology for problem-solving, but it generally does not reach the severity associated with addiction” (page 3). In a number of papers the concept of dependence/dependency is defined referring to the terms “reliance”/“overreliance” (A09; A11; A17; A19; A24; A26; A32; A34), for example A11 defines AI dependency as “an excessive reliance on AI technologies and applications across various aspects of life, including academic studies, daily routines, and social interactions” (page 3); in three more papers (A08; A13; A31) the two terms are clearly used as synonyms, but none of the terms were defined. To a lesser extent, dependence was also defined in terms of ‘compulsive use’ (A01; A22; A33).

A37 targets reliance and uses the same term in its definition, but in fact refers to emotional bonding. Those authors who refer to addiction as the target of their studies remain consistent in its definition (A03; A15; A25; A27), even though “psychological dependence” is sometimes included (A27; A15). Likewise, authors who state that they examine compulsive use adopt that label consistently (A30; A05; A36).

A certain number of papers generically state to investigate problematic AI use (A06; A10; A14; A21; A29), a concept that is, in some of them, further specified afterwards: A10 refer to dependence, while A29 to compulsive use, A06 describes problematic AI use as an “excessive and maladaptive chatbot use behavior” (page 2), A14 refer to academic dishonesty, such as plagiarism and cheating, finally A21 and A36 leave the issue undefined.

Overall, although the definitions often overlap and highlight different aspects of problematic AI use simultaneously, a bottom-up analysis highlighted that these studies can be grouped into three broad strands: (1) studies that adopt the framework of behavioral addictions and/or focus on compulsive use; (2) studies that consider cognitive (over)reliance on AI systems, such as replacing personal skills and capabilities with AI; and (3) studies that consider the psychological and emotional dimension of dependence, e.g., the formation of parasocial relationships with AI systems. Note that strand (1) refers to a theoretical model of problematic AI use, while strands (2) and (3) are model-agnostic dimensions of interest that can co-occur. In a few additional cases, authors did not provide an explicit definition of problematic AI use and were therefore classified within the “undefined” strand. Finally, some studies might appear to fit more naturally within a different strand than the one to which they were assigned; this is motivated by the conceptualization provided by the authors. For instance, A18 and A27, which examine psychological and emotional dependence, were classified within the behavioural addiction strand because of the specific conceptualization of problematic use adopted by the authors (see Table 2).

**Table 2:** How “problematic AI use” is considered in the selected articles.

Strand	Studies
Behavioral addiction and/or compulsive use	A01; A03; A05; A07; A09; A10; A18; A22; A23; A25; A27; A28; A29; A30; A33; A36
Cognitive (over)reliance	A11; A14; A15; A17; A19; A24; A26; A32; A34
Psychological and emotional dependence	A02; A04; A06; A15; A28; A37
Undefined	A08; A12; A13; A16; A20; A21; A31; A35

#### 3.3.1 Behavioral addiction and/or compulsive use

In many studies, problematic AI use is considered in terms of addiction or habituation, with a focus on observable behaviors rather than emotional states, and often explicit adoption of the behavioral addiction framework. In some studies, AI dependency has been described as a specific form of behavioral addiction (A23; A25), defined by excessive involvement in otherwise acceptable activities when practiced in moderation (A23), and distinguished from other forms of internet or short video addiction (A28). In A07, the broader concept of technological dependence is used, referring to intensive use of tools and services essential for daily activities and goal achievement. Within the framework of digital technology addiction, chatbot dependence is often described as a repetitive behavior that disrupts normal functioning, characterized by salience, tolerance, and withdrawal (A10; A18), with both behavioral and relational components (A27). Although “behavioral addiction or dependence” is frequently invoked, there is currently no official clinical diagnosis for addiction to social chatbots (A18). Nevertheless, some researchers have explicitly drawn on the DSM-5 or DSM-5-TR framework (American Psychiatric Association, 2013, 2022), applying its criteria for behavioral addictions to generative AI technologies (A09; A25); others have adapted definitions and criteria from problematic Internet, social media, and gaming use to better understand problematic behaviors related to ChatGPT (A10). In other cases, studies highlight the compulsive use as an extension of the behavioral perspective, characterized by persistent and often uncontrollable impulses toward AI.

Researchers have situated excessive and compulsive AI use within the broader context of digital technologies, including social media and online gaming (A29). Others have more precisely conceptualized compulsive behavior in AI use as a persistent need to engage with AI tools to complete tasks or solve problems (A01). For instance, compulsive use of ChatGPT has been defined as excessive and frequent engagement with the platform, accompanied by cognitive preoccupation, increased tolerance requiring more frequent use to achieve satisfaction, withdrawal symptoms when access is restricted, and an inability to stop using ChatGPT (A03; A05; A36). In the case of social chatbots, compulsive chatting has been described in A30 as intensified human–machine interaction that interferes with other areas of daily life. Among studies that conceptualize problematic AI use as compulsion, this form of use is seen as extending the original concept of internet addiction to a wider range of potentially harmful interaction patterns. Emerging descriptions of compulsive behaviors such as extensive use of ChatGPT to converse or seek advice on personal life issues are viewed as adaptations to the evolving digital landscape (A05).

#### 3.3.2 Cognitive (over)reliance

Several studies (see Table 2) have addressed the issue of problematic AI use in terms of cognitive reliance and over-reliance. Notably, while reliance on AI is not inherently problematic, some studies still characterized it as a form of AI dependency without using the more precise term overreliance. In both cases, problematic AI use is described as excessive functional dependence on AI tools linked to the systematic replacement of activities with AI, which can impair autonomy and critical thinking. Specifically, in this context AI dependency has been defined as excessive reliance on AI technologies and applications across multiple aspects of life, such as academic studies (A11), or as a high level of trust in particular technological tools and services, to the point that they become indispensable for completing daily activities and achieving goals (A24; A34). Particular attention has been given to its use in educational settings: indeed, over-reliance in education may affect students’ ability to solve problems independently (A17; A34). Another concern is that users may blindly trust the incorrect information provided by generative AI and thus make poor decisions. Furthermore, over-reliance on generative AI could undermine creativity and problem-solving skills, leading to a loss of independent thinking and judgement (A15). Lastly, some studies also frame the problematic AI use in terms of improper academic behaviors, such as plagiarism and cheating when using ChatGPT (A14), a deliberate use that straddles the line between behavior-focused definition of problematic AI use and cognitive (over)reliance on AI.

#### 3.3.3 Psychological and emotional dependence

A37 highlights that, while research on technology addiction has often focused on behavioral patterns of use, emerging evidence points to the need for a more nuanced understanding that considers the psychological and emotional dimension of relationship with technology. In A15, generative AI addiction is compared to social media addiction and defined as a psychological state in which individuals develop an excessive attachment to AI systems and find it difficult to stop using them. Beyond behavioral manifestations, researchers have also emphasized the role of underlying psychological mechanisms contributing to problematic AI use and addiction (A28). A key aspect discussed in the literature concerns the emotional experience associated with the absence of technology. A37 describes a feeling of “incompleteness” when users are unable to access AI systems, suggesting a form of emotional attachment that goes beyond merely utilitarian motivations. This sense of discomfort or anxiety in the absence of technology has been interpreted as evidence of a deeper emotional bond, in which AI tools become psychologically significant to users’ sense of self or are used as coping strategies. Drawing on research on human–robot interaction, some selected research have also explored emotional dependence on AI systems such as ChatGPT, characterized by spending increasing amounts of time interacting with the system, perceiving it as essential to one’s daily life, and experiencing negative emotions when access is restricted (A02; A04), thus overlapping with the behavioral strand described above, though with a focus on emotional states. A06 also refers to “Problematic AI Chatbot Use,” defined as the use of AI-based chatbots in which individuals develop an emotional attachment to AI-based chatbots and exhibit problematic interaction patterns.

#### 3.3.4 Undefined

Finally, some studies did not provide an explicit definition of problematic AI use (A08; A12; A13; A16; A20; A21; A31; A35).

### 3.4 RQ 2—how is problematic AI use measured?

The existing literature predominantly employs cross-sectional designs, with quantitative (30 studies) or mixed-method (7 studies) approaches. The range of tools adopted is heterogeneous and includes 1) self-developed tools—which may or may not be validated in the context of the study—2) tools specifically validated for AI-related dependence, as well as 3) tools resulting from the adaptation of scales originally designed for other technologies (such as computers or social media) or finally, 4) tools for different kinds of dependence without any adaptation.

A certain number of studies are based on self-developed tools (A04; A08; A14; A17; A18; A24; A33; A34; A35; A37), some of which are validated using factor analysis within the study itself (A08; A14; A24).

Within the analyzed corpus of papers, five studies were identified as explicitly aimed at validating a newly developed scale. Specifically, A19 validated a LLM dependence scale (LDS) that distinguishes between 1) functional dependence, which refers to trust in the content generated by LLMs, and 2) existential dependence, addressing the symptoms characterizing addiction (withdrawal, tolerance, impairment of functionality). Notably, in the broader addiction literature, functional dependence denotes dependence that impairs functioning, a feature here captured by the “existential dependence” factor, while the “functional dependence” factor seems to measure non-impairing reliance.

A10 validated the Problematic ChatGPT use Scale that was developed based on the framework of Internet Gaming Disorder in the DSM-5-TR (American Psychiatric Association, 2022). This scale was used in one other paper included in the review (A29).

A32 validated the AI Chatbot Dependence Scale. Authors identified potential items from previous publications (i.e., the scale of intelligent machines dependence (Tang et al., 2023), AI dependence (A02), other technology dependence scales (Igarashi et al., 2008; Jenkins-Guarnieri et al., 2013; Elphinston and Noller, 2011; van den Eijnden et al., 2016; Trub and Barbot, 2016), and in-depth interviews.

A09 validated the scale of Dependence on AI, incorporating criteria adapted from compulsive behaviors or dependencies described in DSM-5 (American Psychiatric Association, 2013). These criteria were broken down into five key components: (1) Feeling of vulnerability; (2) Concern about relevance and performance; (3) Need to maintain an updated image; (4) Seeking external validation; and (5) Fear of personal obsolescence. This last scale was adopted in some of the studies analyzed for the review (A01; A22; A23; A26; A29).

The fifth study (A31) is the only validating a scale for AI (over)reliance in the specific fields of reading, writing and numeracy/arithmetic.

Some scales were adapted for AI dependence but were originally built for other dependences. Table 3 reports the original scales and in which studies they have been adapted and used.

**Table 3:** Scales for other dependences adapted to the AI.

Type of scale	Specific scale	Studies
Scales for social media	1) Bergen Addiction Scale (Andreassen et al., 2012) 2) Social media addiction scale (Cao et al., 2020)	1) Adapted in A21, used in A11, A20, A28. 2) Adapted to become the Generative AI Addiction (GAA) scale and used in studies A15 and A25.
Scales for mobile applications	The Compulsive use of mobile applications (Clements and Boyle, 2018) and the Compulsive social app usage (Hsiao et al., 2017), that was in turn adapted from Lee et al. (2014)	Adapted in A05, used in A36
Scales for smartphone	Smartphone Addiction Scales (Kwon et al., 2013).	Adapted in A02 as AI Dependence Scale
Scales for computer	1) Scale for computer addiction (Charlton, 2002) 2) Scale for computer addiction (Charlton and Danforth, 2010) in turn adapted from Computer Apathy and Anxiety Scale (CAAS) (Charlton and Birkett, 1995).	1) Contributed to the scale developed in A04 and to the AI Addiction scale developed in A03 2) Contributed to the AI Addiction scale developed in A03
Scale for fast food	Scale for fast food addiction (Farah and Shahzad, 2020).	A27 included 7 items

Regarding the last category, we found that A30 uses the scale developed by Panda and Kumar Jain (2018) without reporting any adaptation.

An important point is that in some cases the developed scales are adopted and used for different definitions of AI dependence than the one for which they were originally validated. This can result in a mismatch between the constructs the study purports to measure and the way these constructs are operationalized in questionnaire items. To further examine this aspect, where available, we systematically re-examined the item content used in each study and recoded measures based on their substantive alignment with the target construct.

For example, A07 frames “AI dependence” primarily as in behavioral terms applied to a learning context, yet it adopts a scale which operationalizes the construct differently. Operationally, the indicators blend two distinct measurement approaches: (a) behavioral-addiction/loss-of-control items (e.g., “I often use ChatGPT involuntarily,” “I find it difficult to stop using ChatGPT”) and (b) psychoemotional attachment/anthropomorphizing items (e.g., “ChatGPT is like an intimate friend,” “I would feel uncomfortable without ChatGPT,” “My life is filled with conversations with ChatGPT”). This hybrid item set is only partly behavioral, and likely captures a blended latent construct—compulsive use, emotional/parasocial attachment, and in part even high-frequency preference (e.g., “I always prefer to use ChatGPT to find information,” which may reflect perceived usefulness rather than impairment)—thereby presenting a gap between what the authors claim to measure and what their items actually index.

Relatedly, A22 employed a dependency scale combining items on workplace use, behavioral addiction, and AI anxiety, while also using correlate variables with substantial item overlap with the outcome variable (e.g., AI anxiety).

Table 4 reports our own assessment of which type of AI problematic use is captured by item content, crossed with the conceptualization of problematic AI use adopted in the study.

**Table 4:** Conceptualization of problematic AI use *vs* operationalization.

operationalization → conceptualization↓	Behavioral addiction and/or compulsive use	Cognitive (over)reliance	Psychological and emotional dependence	Hybrid/undefined/not retrievable
Behavioral addiction and/or compulsive use	A05; A10; A18; A28; A29; A30; A36;	A01; A33	A18; A27	A03; A07; A22; A23; A25
Cognitive (over)reliance	A11; A15; A24	A17; A34	—	A19; A26
Psychological and emotional dependence	A02; A06; A15; A28	—	A04	A37
Undefined	A16; A21	A13; A20	—	A35

Taken together, the operationalisation problems noted above reflect three recurring weaknesses. First, several scales conflate analytically distinct dimensions within a single score, e.g., A07’s mixing loss-of-control items with psychoemotional and anthropomorphising items. Second, item content is often only loosely aligned with the construct the scale claims to measure, e.g., A19 labels as “functional dependence” a factor that largely reflects trust in AI output, while A22’s dependency items overlap substantially with AI anxiety. Third, instruments validated for one conceptualisation are reused for another (e.g., the scale validated in A09), so that nominal comparability across studies can mask construct drift. Underlying all three, most scales import their criteria wholesale from behavioural addiction frameworks (A09; A10; A32), tending to presuppose rather than test that problematic AI use shares the structure of gambling, gaming, or social-media use, leaving the AI-specific dimensions identified here under-measured.

### 3.5 RQ 3—which correlates and associated outcomes have been investigated for each definition of problematic AI use?

To map what correlates were studied in relation to problematic AI use, we start from the categorization developed in the RQ 1, i.e., by grouping studies in three research strands: those focusing on behavior and/or compulsive use (often within a behavioral addiction framework), those considering cognitive (over)reliance, and those focusing on psychological and emotional dependence. Note that three scale-development and validation studies (A09, A31, and A32) and A14 are not discussed in this section, as they did not investigate substantive correlates of problematic AI use.

#### 3.5.1 Behavioral addiction and/or compulsive use

Fifteen studies in this category (A01; A03; A05; A07; A10; A18; A22; A23; A25; A27; A28; A29; A30; A33; A36) studied correlates of this phenomenon. The variables examined partly mirrored those investigated in the broader literature on digital and behavioral addictions, such as age (A01), gender (A10), personality traits (A29; A36), anxiety (A05), burnout (A05), and general distress (A29).

Interestingly, some studies considered variables related to AI itself, such as AI-related anxiety (A01; A22; A23; A33) and metacognitive beliefs about generative AI (both positive and negative) (A25).

On the other hand, many studies focused on use in academic settings and, as such, considered variables related to that context, such as user satisfaction with ChatGPT (A07; A10), academic stress (A33), awareness of innovation (A03), performance expectations (A33), and intention to delegate (A07). AI dependence and compulsive use were considered in connection to academic self-efficacy and, indirectly, to concerns about autonomy and critical thinking (A33; A01; A22), and A03 linked AI dependence with tolerance for academic cheating.

Outside of the academic context, studies focused on affective predispositions, considering variables such as parental and peer phubbing, loneliness, fear of social judgement, and self-efficacy (A30; A28; A27) or, in the case of social chatbot dependence, loneliness, trust in the chatbot, and its anthropomorphizing (A18). Other work investigates loneliness and fear of social judgement as correlates of AI dependence (A30; A27). A27 also considered perceived characteristics of social chatbots themselves, i.e., perceived sentience, availability and warmth of Replika.

#### 3.5.2 Cognitive (over)reliance

Seven studies (A11; A15; A17; A19; A24; A26; A34) examined the relation between AI dependence and other variables while conceptualizing AI dependence as cognitive (over)reliance.

Of these, three study the association between AI literacy and cognitive (over)reliance on AI (A19; A24; A17), and one examines the impact of conceptualizing competency in using LLMs as part of one’s own identity (A19). Academic stress (A11) and trust in AI (A17; A24) were also investigated as potential correlates of overreliance.

On the side of AI characteristics that impact problematic AI use, A15 examined anthropomorphizing, interactivity, intelligence, and personalization, via the mediation of flow and attachment.

In terms of consequences, two studies (A17; A24) investigated AI overreliance’s impact on 21st-century skills such as problem solving, critical thinking, creative thinking and collaboration. A26 examined its impact on research skills, research disposition, and self-efficacy in academia. Finally, A34 examined the impact on self-efficacy.

#### 3.5.3 Psychological and emotional dependence

Six studies focused on psychological and emotional dependence (A02; A04; A06; A15; A28; A37). A02 tested cross-lagged links between adolescent mental health problems and AI dependence, with distinct usage motivations (escape, social, and entertainment, instrumental) as potential mediators. A37 related technical savviness to AI reliance patterns and emotional reactions to technology absence. A04 applied the triangular theory of love to ChatGPT interactions, linking perceived emotional intelligence and companionship affordances to intimacy, passion, commitment, and ultimately emotional dependence, with anxious attachment as a moderator. Lastly, A06 connected emotional dependence with low self-esteem, social anxiety, escapism, and flow.

Complementing these user–AI relational perspectives, A28—with a conceptualization blending psychoemotional features into a behavioral addiction framework—adopted a socio-ecological approach and investigated the impact of parental and peer phubbing in fostering AI dependency. Finally, A15 used a Cognition–Affect–Conation framework, linking cognitive perceptions (anthropomorphizing, personalization) to affective mechanisms (flow, emotional attachment).

#### 3.5.4 Undefined

The remaining studies, which do not provide a formal definition of AI dependency, primarily examine how psychological correlates, cognitive mediators, and behavioral outcomes combine to explain why some users develop problematic reliance on AI (A08; A12; A13; A16; A20; A21; A35). Key independent variables in this subset often include maladaptive personality traits and negative emotional states, including them in complex mediation models; A20 considered neuroticism, self-critical perfectionism, and impulsivity, with needs frustration, negative academic emotions, and positive performance expectations as mediators. Relatedly, A21 studied social anxiety and low self-esteem. On the cognitive and behavioral side, A35 examined motivation and self-reliance as correlates of students’ dependency. On the academic side, A08 highlighted that dependent participants questioned their own skills when not allowed to use AI, while impact on actual skills was examined with contrasting results (A12, A13). Lastly, with respect to mental health and downstream outcomes, A16 studied the relationship between AI chatbot dependency and depression.

### 3.6 RQ 4—what preliminary evidence is available on correlates and associated outcomes for each operationalization of problematic AI use?

As reported under RQ2 results, some studies present a mismatch between the AI dependence definition and conceptualization they focus on, and the operationalization offered by the scales they employ.

When analyzing RQ3, which was a simple mapping of variables investigated, we grouped studies according to the authors’ definitions of problematic AI use. In the case of RQ4, instead, we re-coded all studies according to the actual operationalization of problematic use emerging from analysis of the scales used, as we believe an examination of results necessitates a critical analysis of what aspects of problematic use are actually investigated. Accordingly, results for this Section refer to the categorization of operationalizations reported in Table 4, rather than the definitions or conceptualizations presented or adopted by the authors.

While this does not assuage validation concerns (such as when a scale for another behavioral addiction is adapted to AI use without re-validating it), it can still aid in interpreting results.

#### 3.6.1 Behavioral addiction and/or compulsive use

Thirteen studies measured addiction by focusing on behavioral cues (e.g., frequency of use) or by adapting scales for other behavioral addictions, such as social media addiction (A02; A05; A06; A10; A11; A16; A15; A21; A24; A28; A29; A30; A36).

Most of these studies focus on correlates for AI behavioral addiction or compulsive use that go from addiction symptoms to sociodemographics. Depression emerged as the most consistently investigated correlate, with four studies examining its relationship with this phenomenon. Across these studies the correlation was consistently positive but varied considerably across samples. While the positive association appears robust, the direction of causality remains unclear. It should be noted that A29 computed the Depression, Anxiety and Stress Scale (DASS; Lovibond and Lovibond, 1995) score as if it were unifactorial, rather than deriving separate scores for depression, anxiety, and stress as the scale’s scoring procedures require, potentially affecting the reliability of its results.

Social distress indicators constituted a second category of correlates. Loneliness, as measured by the UCLA Loneliness Scale (A02), was associated with AI dependence, and so did social anxiety across three studies (A06; A21; A30) employing different measurement scales. Additional socially-related correlates included fear of negative evaluation (A30) and parental phubbing (A28), the experience of feeling ignored by parents due to their smartphone usage.

Studies investigating these social distress correlates also explored potential coping mechanisms that might mediate the relationship between social difficulties and problematic AI use. Escapism emerged as a correlate in two studies (A02; A06). Additionally, experiencing flow states during AI use was associated with problematic chatbot use and AI addiction (A06; A15).

Finally, negative self-perceptions appeared to increase vulnerability to AI dependence. Both low self-efficacy (A28) and low self-esteem (A06) were associated with problematic behavioral patterns. A11 found that academic stress mediates this relationship. Compulsive AI use is also linked to other internet and gaming addiction (A29).

Four studies examined problematic AI use in relation to personality traits and socio-demographic characteristics (A01; A10; A29; A36). Across this small body of evidence, younger age and male gender consistently emerged as significant correlates of problematic use (A01; A10; A36). With respect to personality, higher conscientiousness and agreeableness appear to function as protective factors (A29; A36), whereas neuroticism is associated with problematic AI use (A36).

Other studies have identified additional cognitive and motivational correlates of problematic AI use. Specifically, higher performance expectations regarding AI tools, namely, the belief that AI will substantially enhance one’s outcomes or efficiency, have been associated with elevated AI dependency levels (A11). In parallel, lower critical thinking or weaker tendencies to evaluate outputs skeptically and independently appears to be linked to greater AI dependency vulnerability (A24).

Emerging evidence also points to potentially deleterious outcomes associated with problematic AI use. In A24, higher AI dependency was related to poorer functioning in domains such as creativity, problem solving, and collaboration, alongside lower self-esteem. Compulsive ChatGPT use has been associated with burnout, as well as with heightened anxiety and sleep disturbances (A05). Importantly, these findings are largely correlational, and directionality is not yet established: for instance, anxiety or burnout could both contribute to, and be exacerbated by, AI-dependent coping and work habits.

#### 3.6.2 Cognitive (over)reliance

Six studies operationalized problematic AI use not through addiction-derived criteria but specifically as cognitive (over)reliance (A01; A13; A17; A20; A33; A34). Correlates for cognitive (over)reliance emerged across multiple domains.

Academic stress seems to play a role, as A20 identified negative academic emotions to be indirectly associated with overreliance on AI, mediated by performance expectation. The same study identified basic psychological need frustration (in terms of autonomy, relatedness, and competence) to be both directly and indirectly associated with overreliance. Lastly, this study also considered a longer causal pathway, identifying that neuroticism, self-critical perfectionism, and impulsivity may be at the root of these negative emotions and need frustration.

Surprisingly, two studies identified academic self-efficacy as a correlate (A33; A34). However, A34 conflated the constructs of academic self-efficacy and perceived AI effectiveness. A33 operationalized the construct adequately but still presents the counter-intuitive result that self-efficacy and self-reported critical thinking correlate with overreliance.

Users’ perceptions of and attitudes toward AI systems constituted another important category of correlates of (over)reliance. Among mathematics teachers, AI literacy and trust in AI were associated with reliance (A17). Performance expectation from AI was a significant correlate of overreliance in A20, while A33 did not confirm this effect.

In terms of consequences of overreliance, studies focused predominantly on cognitive and skill degradation. One quantitative study among mathematics teachers documented negative impacts of reliance on AI across multiple professional competencies: collaboration, communication, problem-solving, creativity, critical thinking, and self-confidence (A17). Experimental evidence further corroborated these patterns: students who had been working with AI assistance demonstrated notable performance decrements in problem-solving and coding tasks when AI tools became unavailable (A13).

#### 3.6.3 Psychological and emotional dependence

Eight studies examined psychological and emotional dependence on AI (A02; A04; A06; A15; A18; A27; A30). However, only three (A04; A18; A27) specifically operationalized problematic AI use through an emotional-relational attachment lens, at least in their qualitative measures. The remaining five adopted addiction frameworks despite examining attachment-related phenomena, an inconsistency that illustrates the construct-operationalization gap discussed above.

The three studies employing emotional-relational operationalization identified correlates primarily within the socio-emotional domain. Loneliness emerged as a consistent correlate (A18; A27), alongside social anxiety (A27) and anxious attachment style (A04).

These studies uniquely examined how AI system characteristics might foster problematic AI use. Constant availability was identified as a potentially important factor, though findings were inconsistent across studies (A04; A27). More consistently, users’ perceptions of AI characteristics emerged as significant correlates: perceived emotional intelligence (A04), sentience (A27), warmth (A27), and trustworthiness (A18) were all associated with problematic AI use patterns. This pattern extended to anthropomorphizing tendencies (A18) and sense of relationship with the AI (A27) serving as correlates. Notably, A27 found that users who felt socially evaluated by AI systems—experiencing discomfort about being “judged” by the technology—showed increased problematic AI use. Additionally, deriving well-being from AI interactions was associated with problematic AI use (A27).

A18 provided a conceptual bridge between attachment and addiction frameworks: while operationalizing problematic AI use as emotional attachment, it documented outcomes characteristic of behavioral addictions, including salience, tolerance, and withdrawal symptoms.

## 4 Discussion

A preliminary examination of the geographical and contextual distribution of the included studies is necessary to adequately contextualise the current state of the field. Our findings reveal a marked geographical imbalance, with almost half of the studies originating from China (17 studies), followed by smaller groups in Peru (4) and the Philippines (3). This distribution suggests that the current evidence base is significantly influenced by specific cultural and institutional contexts, particularly in East Asia and South America, whilst other regions are under-represented. This concentration in China raises the question of whether it reflects a more pronounced manifestation of the phenomenon in that context, or rather differences in the maturity of research across countries, where the study of such dynamics may still be in its infancy.

From a contextual perspective, the literature focuses predominantly on academic settings, with over half of the studies (20 out of 37) involving university students. Consequently, the current profile of problematic AI use, derived largely from studies conducted on university populations, particularly in China, should be interpreted with caution. It remains to be determined whether the correlations identified, such as academic stress and performance expectations, represent generalisable characteristics of the phenomenon or are partly influenced by high-pressure educational environments and the rapid integration of AI, which are typical features of such contexts.

Beyond these geographical and contextual imbalances, the findings from RQ1 further highlight a significant terminological and conceptual heterogeneity within the literature. Terms such as “addiction,” “dependence,” “dependency,” “overreliance,” and “compulsive use” are employed with varying degrees of precision, sometimes treated as synonyms and sometimes explicitly distinguished. A label that is used across the literature is dependence/dependency, which is associated with multiple usage patterns that range from reliance to parasocial bonding. This places under the same “umbrella” term a variety of behavioral, cognitive, and psychological expressions that would warrant a clearer distinction. This terminological inconsistency might be a mere labelling problem, an early-stage fragmentation typical of emerging research fields. However, we argue that the issue runs deeper: the conceptual heterogeneity observed in the literature reflects unresolved theoretical tensions regarding the very nature of the phenomenon under investigation.

This is most evident in the widespread practice of importing definitions and diagnostic criteria from the behavioral addiction literature. Several studies explicitly draw on DSM-5 or DSM-5-TR criteria (American Psychiatric Association, 2013, 2022) for behavioral addictions, operationalizing problematic AI use through constructs such as tolerance, withdrawal, salience, and loss of control. This approach is questioned for the AI by Ciudad-Fernández et al. (2025), and more in general by Kardefelt-Winther et al. (2017), since often the presence of the above-mentioned symptoms does not reflect a real functional impairment in individuals’ lives, unlike in more severe and disabling addictions, like substance use disorders. Moreover, while this approach offers the advantage of anchoring a new phenomenon to an established clinical framework, it also carries significant epistemological costs. The criteria for behavioral addiction were developed for phenomena in which the “stimulus” is relatively passive or unidirectional, while, as Kooli et al. (2025) also highlight, AI entails an active engagement process. Gambling machines do not adapt to the user’s emotional state; the internet does not simulate empathy; social media feeds, while algorithmically curated, do not engage in reciprocal conversation. Generative and conversational AI systems may be qualitatively different. They respond, adapt, and, crucially, simulate understanding. They can be designed to provide unconditional validation, to mirror user opinions, and to avoid the friction inherent in authentic human interaction.

By adopting a behavioral addiction framework without modification, researchers risk rendering certain dimensions of problematic AI use invisible to their own instruments. The criteria of tolerance and withdrawal, for instance, may capture some aspects of compulsive use, but they are ill-suited to detect phenomena such as the preference for AI interaction over human connection^1^.

This theoretical blind spot may help explain an emerging gap between public discourse and academic research^2^. Media coverage of problematic AI use has increasingly focused on relational and emotional dimensions^3^: reports of users developing romantic attachments to chatbots, concerns about social withdrawal among young people who prefer AI companions to human friends, and debates about the psychological effects of AI sycophancy. At the extreme, popular press coverage has raised concerns about so-called AI-induced psychosis. Yet our review indicates that the emotional dimension remains under-investigated in the empirical literature. Studies examining the emotional and psychological dimension of problematic AI use are relatively scarce (eight among considered articles, of which five using the addiction framework). This might not be simply a matter of research priorities; it may reflect the lack of validated scales capable of measuring relational and emotional attachment to AI systems. And this lack, in turn, might stem from the widespread adoption of definitional frameworks that do not foreground these dimensions.

We do not suggest that behavioral addiction criteria are irrelevant to understanding problematic AI use. Indeed, several studies in our review provide evidence of tolerance, withdrawal, and compulsive patterns among AI users. Rather, our argument is that an exclusive or primary reliance on these criteria produces a systematically incomplete picture of the phenomenon. Indeed, the analysis of how problematic AI use was conceptualized in the corpus of papers drew us to identify three main research strands which reflect its complexity: studies focusing on behavior and compulsive use, on cognitive (over)reliance, and on psychological and emotional dependence. These three strands reflect distinct perspectives on problematic use, while also offering complementary insights and potential points of interaction. The first of these typically includes studies that explicitly adopt the behavioral addiction framework, while the other two can be more aptly described as dimensions of problematic AI use a study can focus on, rather than theoretical models. The first strand draws on theoretical models developed for technology and substance addiction and focuses on specific patterns of use and symptoms of addiction, like salience, tolerance or withdrawal. The second strand, the literature on cognitive overreliance, focuses on the excessive delegation of cognitive tasks, judgments, or decision-making processes to AI systems and related consequences. This strand is more clearly defined, with well-delimited boundaries, and therefore shows less overlap with the other two. This is probably due to the fact that studies included are related almost exclusively to the academic context. The third strand addresses the psychological and emotional dependence literature, meanwhile, examines users’ affective bonds with AI technologies. Although it should be situated within the broader field of relational dependence research, it seems to be frequently connected with the first strand. This is particularly evident in the studies in our corpus where conceptualizations and operationalizations appear inconsistent; for example, some studies conceptualize the phenomenon as a form of psycho-emotional dependence while measuring it with scales derived from a behavioural addiction framework.

Given the debated use of the addiction framework for investigating the excessive or maladaptive use of technologies (Kardefelt-Winther et al., 2017) and the richness brought by complementary approaches, a more adequate conceptualization would need to integrate both the cognitive and relational dimensions, which are analytically distinct but empirically intertwined, and the behavioral addiction framework may hinder this integration.

Lastly, it is important to avoid pathologising any form of reliance on AI. The broader literature on Internet and technology use cautioned about this kind of risk when defining addiction (Kardefelt-Winther et al., 2017). In fact, much of what might be labelled “AI dependence” may reflect adaptive delegation: the productive offloading of routine cognitive operations that can augment rather than degrade performance and is functionally analogous to long-accepted reliance on calculators, search engines, or external memory aids. What distinguishes problematic from non-problematic use is therefore not the presence of reliance, habitual engagement, or even attachment per se, but whether these produce genuine functional impairment, loss of control, or distress. This distinction is hard to draw in several of the reviewed studies, whose instruments index frequency or preference (e.g., “I always prefer to use AI to find information”) rather than impairment, thereby conflating efficient, augmentative use with dependence. Future conceptualisations should accordingly locate problematic AI use on a continuum that explicitly preserves a category of beneficial, augmentation-oriented reliance against which genuinely maladaptive patterns can be identified, especially as industry and society adopt AI as a standard tool for productivity and everyday life, as it happened with the Internet and with smartphones.

### 4.1 Correlates of problematic AI use

Several interpretive patterns can be drawn from the mapped evidence, although they should be treated as provisional given the heterogeneity of definitions and measures.

First, the cluster of social-distress correlates loneliness (A02), social anxiety (A06; A21; A30), fear of negative evaluation (A30), and parental phubbing (A28) converges on the hypothesis that problematic AI use may partially originate from unmet social needs, a theme that also reappears in the emotional–relational attachment strand. Within this framework, escapism (A02; A06) can be interpreted as an avoidant coping pathway, while flow (A06; A15) may sustain engagement and facilitate escalation.

Second, negative self-perceptions (such as low self-efficacy A28) and low self-esteem (A06) appear to increase vulnerability, A11 suggests that academic stress may mediate these links, with performance expectations playing an important direct role. Lower critical thinking (A24), low self-esteem (A06), and higher performance expectations (A11) may compound and lead to compulsive or over-reliant use due to the AI’s output being perceived as better than one’s own.

In the cognitive (over)reliance strand, the counterintuitive finding that academic self-efficacy (and even self-reported critical thinking) is associated with overreliance (A33; A34) may reflect measurement overlap (e.g., conflating self-efficacy with perceived AI effectiveness in A34 or the possibility that more confident users feel more comfortable delegating tasks.

The fact that AI literacy and trust correlate with reliance on AI among mathematics teachers (A17) suggests a “familiarity/trust paradox,” where greater competence and confidence may increase vulnerability to excessive delegation rather than protect against it. Convergent evidence on performance decrements when AI becomes unavailable (A13) is consistent with the notion that repeated delegation can impair independent skill execution.

Overall, the literature is compatible with a potentially self-reinforcing cycle: users adopt AI to enhance performance, develop positive expectations that foster dependency, and ultimately experience degradation in the very competencies AI was intended to augment yet key anomalies (e.g., the self-efficacy findings) require replication and stronger designs.

Finally, within the psychological and emotional dependence strand, the typical user profile again points to unmet social needs, but also uniquely highlights the role of perceived AI characteristics as correlates of problematic AI use; notably, users’ discomfort about being socially evaluated by AI and deriving well-being from AI interactions (A27) suggest a plausible pathway from adaptive coping to maladaptive dependence.

A18 further bridges attachment and addiction frameworks by showing addiction-like outcomes (salience, tolerance, withdrawal) despite operationalizing problematic AI use as emotional attachment, implying that boundaries between frameworks may be empirically blurrier than current practices assume; at the same time, downstream consequences of emotional attachment (e.g., effects on offline relationships, social skills, well-being) remain largely unexamined beyond the addiction-like symptoms reported in A18. In this context, Attachment Theory provides a valuable framework for interpreting these findings (Bowlby, 1969; Main et al., 1985). Indeed, problematic use of AI may arise from the human need to seek secure attachment figures in response to situations of emotional vulnerability, such as loneliness (A18, A27). The specific properties of AI, such as its constant availability and non-judgemental responsiveness, enable it to act as a ‘relational surrogate’. Unlike the complexities and tensions inherent in genuine human relationships, AI can therefore be perceived as a “safe haven,” particularly by individuals with a history of insecure attachment and insecure internal working models (A04).

## 5 Conclusions and future directions

### 5.1 Toward integration: cognitive and emotional dimensions as interrelated phenomena

The tripartite distinction we have employed throughout this review behavioral addiction and/or compulsive use, cognitive (over)reliance, and emotional attachment has proven useful for organizing a heterogeneous literature in three main strands. However, it is worth considering whether these categories, while analytically distinct, may be more empirically interrelated than our grouping would suggest.

A suggestive pattern emerges from the studies in our review. Research examining cognitive (over)reliance in academic or professional contexts frequently identifies emotional correlates: academic stress, negative emotions, and psychological need frustration appear as correlates of what is ostensibly a cognitive phenomenon. This cognitive-emotional interaction can be understood within the framework of Self-Determination Theory (SDT; Deci and Ryan, 2000), according to which well-being depends on the satisfaction of the basic needs for autonomy, competence, and relatedness. The findings suggest that the frustration of these needs, often driven by academic stress or social isolation, may be directly associated with excessive reliance on AI. In this context, AI may function as a dysfunctional compensatory mechanism: delegating tasks to the system provides an immediate sense of capability and productivity, while its constant availability and responsiveness help reduce feelings of loneliness, offering instant gratification that compensates for deficits in real-world social relationships. Complementing this, Uses and Gratifications (U&G) Theory helps explain how individuals actively turn to AI to obtain specific rewards, such as escape, social interaction, or instrumental efficiency, particularly when basic psychological needs are unmet (Katz et al., 1973). By consistently delivering such rewards, AI reinforces repeated use, potentially fostering a shift from functional engagement to habitual and maladaptive reliance. From a cognitive perspective, the systematic delegation of tasks can be interpreted in light of Cognitive Load Theory (Sweller, 1988). Within this framework, excessive reliance may reflect a maladaptive form of ‘cognitive offloading’, an attempt to reduce immediate mental effort. Although this strategy may provide short-term relief, it also limits the cognitive effort required for deep learning, ultimately leading to the decline of skills. This ‘paradox of dependence’ is empirically supported by findings (e.g., A13) showing that individuals who habitually delegate tasks experience a significant decrease in performance when the AI tool is unavailable, as their autonomous problem-solving abilities have been impaired.

At the same time, studies operationalizing problematic AI use through emotional attachment nonetheless document outcomes characteristic of behavioral addictions, including salience, tolerance, and withdrawal. One possible explanation lies in the affordances of AI systems themselves. As noted in our introduction and in light of the considerations discussed within the framework of Attachment Theory, the features that foster emotional attachment, constant availability, non-judgmental responsiveness, and frictionless interaction, may simultaneously facilitate cognitive delegation. An AI system that is always accessible makes it easy to offload tasks; the same characteristics make it a comfortable companion. The phenomenon of AI sycophancy illustrates this dual potential: a system that invariably validates the user’s opinions may both reinforce cognitive biases (a form of overreliance) and create an illusory sense of being understood (a form of emotional attachment).

This perspective aligns with emerging evidence that users who initially adopt AI for purely instrumental purposes may gradually develop emotional engagement patterns without explicit awareness. The trajectory from tool use to relational engagement may be more common than the current literature suggests. If so, measurement scales that focus exclusively on one dimension risk missing important aspects of the phenomenon. A user who reports high levels of functional dependence on AI for academic work may simultaneously be developing emotional attachment patterns that go undetected by scales designed to measure only the cognitive dimension.

We offer this observation as a hypothesis for future investigation rather than a firm conclusion. The evidence base is not yet sufficient to determine whether cognitive and emotional dimensions represent distinct pathways to problematic AI use, co-occurring manifestations of a unitary underlying process, or phenomena that interact and reinforce each other over time. Addressing this question will require new primary studies investigating problematic AI use patterns, as well as measurement scales that capture multiple dimensions simultaneously.

### 5.2 Recommendations for future research

The present review points to several priorities for advancing research on problematic AI use.

First, the field requires greater conceptual precision. Researchers should be explicit about which dimension of problematic AI use they are investigating and should select or develop measurement scales that align with their conceptual focus. The practice of using terms like “addiction,” “dependence,” and “overreliance” interchangeably obscures important distinctions and impedes cumulative knowledge-building. We do not advocate for premature consensus on a single definition, the phenomenon may indeed be multidimensional in ways that resist unification, but we do urge greater clarity about which dimension is under examination in any given study.

Second, there is a pressing need for validated measurement scales specifically developed for AI-related constructs. New instruments should address the three main weaknesses we identified in Section 3.2. Specifically, they should: (i) rely on an explicit construct definition, making sure item content of a single scale does not conflate different manifestations of conceptualisation of problematic AI use; (ii) ensure scale validity, so that it reflects what the instrument purports to measure; (iii) avoid directly adapting scales built for different addictions—while efficient, this method imports assumptions about psychological mechanisms that may not apply to conversational and generative AI. Additionally, new scales should separate use intensity/frequency from functional impairment or distress, so as to not pathologise adaptive reliance. The four validation studies identified in our review represent important steps in this direction, but the scales they produced reflect different conceptualizations and have not yet been widely adopted or cross-validated. Especially needed is evidence of structural validity (confirmatory factor analysis and tests of measurement invariance across populations), of convergent and discriminant validity against adjacent constructs (e.g., AI attitudes, AI anxiety, general technology use), and of criterion validity against indicators of genuine impairment. The development of a measurement consensus, perhaps through systematic comparison of existing scales before further single-study scales proliferate, would substantially advance the field and avoid fragmentation. Judged against these criteria, the current evidence base—which is dominated by self-developed or minimally adapted tools, with the four validation studies identified here (A09; A10; A19; A32) reflecting divergent and only partially overlapping conceptualisations—remains underdeveloped.

Third, the relational and emotional dimensions of problematic AI use warrant greater attention. Studies employing qualitative methods have begun to illuminate these dimensions, but quantitative research lags behind. The development of scales capable of measuring emotional attachment to AI, relational substitution, and related constructs would help close this gap.

Fourth, longitudinal research designs are essential for clarifying causal relationships. The predominance of cross-sectional studies limits conclusions about the directionality of key associations. This limitation is particularly evident in the cross-sectional nature of most studies in our review, which precludes conclusions about whether, for instance, loneliness leads to problematic AI use or whether problematic AI use exacerbates loneliness. A similar issue arises in the cognitive domain, where it remains unclear whether overreliance contributes to skill atrophy or whether individuals with pre-existing skill deficits are more likely to become overreliant. Such questions cannot be adequately addressed with correlational data alone. Future research should therefore prioritize multi-wave longitudinal and panel designs to track user behavior over time. To address the limitations of self-reported data, researchers should incorporate behavioral tracking measures (e.g., interaction logs, prompt frequency, session duration) to obtain more objective indicators of use. Furthermore, the adoption of mixed-method approaches, combining qualitative insights into users’ subjective experiences with quantitative analyses of behavioral patterns, would strengthen causal inference and provide a more comprehensive understanding of the phenomenon. Experimental designs, where feasible, would offer an additional avenue to test causal mechanisms.

Fifth, research should attend more systematically to the role of AI systems’ characteristics in fostering problematic AI use. While several studies in our review examined user perceptions of AI (e.g., perceived warmth, emotional intelligence, trustworthiness), fewer investigated how specific design features such as anthropomorphic cues, sycophantic response patterns, or availability characteristics contribute to problematic AI use patterns. To address these risks, future research should integrate models of ethical AI leadership and socially responsible deployment, such as the Revised Artificial Intelligence Device Use Acceptance (RAIDUA) model (Salam et al., 2026a, 2026b), which emphasizes the role of privacy concerns in moderating user acceptance. Furthermore, design interventions should aim to transition AI from a tool for passive “cognitive offloading” to a partner in active knowledge construction. Frameworks like the Integrated Collaborative Reflection Model (ICRM) (Salam et al., 2026a, 2026b) could provide a roadmap for developing systems that prioritize collaborative reflection and knowledge building over mere task delegation. Understanding these relationships has implications not only for theory but also for the design of AI systems that minimize harm.

Finally, the rapid evolution of AI technology presents a moving-target problem for research. Studies conducted on early chatbot systems may have limited applicability to contemporary large language models, and findings from current systems may not generalize to future developments. Researchers should be attentive to technological context and cautious about overgeneralizing findings across different AI modalities.

### 5.3 Limitations

Several limitations of the present review should be acknowledged. Our search strategy, while systematic, was restricted to two databases (Web of Science and Scopus) and to publications in English. This may have resulted in the under-representation of relevant work published in other languages or indexed in other databases. The inclusion of only peer-reviewed journal articles and conference proceedings excluded grey literature, preprints, and dissertations that may contain relevant findings, particularly given the rapid pace of research in this area. The focus on bibliometric databases may also have systematically excluded perspectives from disciplines that publish through different channels. Anthropological, ethnographic, and humanistic approaches to AI use, which might offer valuable insights into the relational and cultural dimensions of the phenomenon, may be under-represented in our corpus. A more comprehensive understanding of problematic AI use would benefit from interdisciplinary integration that our methodology was not designed to capture.

Furthermore, our review highlights a marked geographical and contextual skew, with the current evidence base predominantly centered on academic populations in specific regions, notably China. This concentration underscores the need for future research to examine problematic AI use across more diverse cultural, professional, and age-related contexts, in order to enhance the generalizability of the identified correlates and underlying mechanisms.

The rapidly evolving nature of the field means that studies published after our search cutoff could not be included. Given the pace of AI development and the surge of research interest in this area, significant new findings may have emerged since our search was conducted.

Finally, the heterogeneity of the included studies in terms of definitions, operationalization’s, populations, and methodologies limited our ability to conduct quantitative synthesis for most outcomes. While we attempted to organize findings according to operationalization type, this approach required interpretative judgments that others might make differently. The patterns we have identified should be treated as preliminary observations that require confirmation through more methodologically homogeneous research.

## Data Availability

The original contributions presented in the study are included in the article/Supplementary material, further inquiries can be directed to the corresponding author.

## Footnotes

1
https://www.theguardian.com/technology/2025/dec/18/artificial-intelligence-uk-emotional-support-research

2
https://www.nytimes.com/2025/06/03/opinion/chatbots-ai-addiction-love.html

3
https://www.nytimes.com/2026/01/26/us/chatgpt-delusions-psychosis.html
