# Supplementary material for: Addicted, attached, or just delegating? A scoping review on “problematic artificial intelligence use”
Source: Front Psychol. 2026 Jul 9;17:1900953. doi: 10.3389/fpsyg.2026.1900953 (PMC13391840; doi:10.3389/fpsyg.2026.1900953)
Supplement: Supplementary file 1 [file Table_1.DOCX]

Table A1 – Results of data charting

| # | Title | Authors (Year) | Country | Study design | Data collection method | Sample | AI tech. |
| --- | --- | --- | --- | --- | --- | --- | --- |
| **A01** | Academic self-efficacy and dependence on artificial intelligence in a sample of university students | Estrada-Araoz et al. (2025) | Perù | Quantitative | Survey | 186 university students | Not specified |
| **A02** | AI technology panic-is AI dependence bad for mental health? A cross-lagged panel model and the mediating roles of motivations for AI use among adolescents | Huang et al. (2024) | China | Quantitative | Survey | 3843 adolescents | Not specified |
| **A03** | Avoid excessive usage: Examining the motivations and outcomes of generative artificial intelligence usage among students | Abbas et al. (2025) | Pakistan | Quantitative | Survey | 394 university students | ChatGPT (Conversat. based on LLM) |
| **A04** | Will users fall in love with ChatGPT? a perspective from the triangular theory of love | Chen et al. (2025) | China | Quantitative | Survey | 466 adults | ChatGPT (Conversat. based on LLM) |
| **A05** | Compulsive ChatGPT usage, anxiety, burnout, and sleep disturbance: A serial mediation model based on stimulus-organism-response perspective | Duong et al. (2024) | Vietnam | Quantitative | Survey | 2602 adults | ChatGPT (Conversat. based on LLM) |
| **A06** | Connecting self-esteem to problematic AI chatbot use: the multiple mediating roles of positive and negative psychological states | Yao et al. (2025) | China | Quantitative | Survey | 563 adults | Chatbot |
| **A07** | Delegation or satisfaction? Explaining AI dependence in college students’ learning | Long et al. (2025) | China | Quantitative | Survey | 293 college students | Not specified |
| **A08** | Dependency on AI-based writing tools in English learning: Implications for human-computer interaction | Zunaidah et al. (2023) | Indonesia | Mixed | Survey and interviews | 148 adults | AI writing tools (e.g., Grammarly) |
| **A09** | Development and validation of a scale for dependence on artificial intelligence in university students | Morales-García et al. (2024) | Perù | Quantitative | Survey | 528 university students | Not specified |
| **A10** | Development and validation the Problematic ChatGPT Use Scale: a preliminary report | Yu et al. (2024) | Taiwan | Quantitative | Survey | 1040 adults | ChatGPT (Conversat. based on LLM) |
| **A11** | Do you have AI dependency? The roles of academic self-efficacy, academic stress, and performance expectations on problematic AI usage behavior | Zhang et al. (2024) | South Korea | Mixed | Survey | 300 university students | ChatGPT (Conversat. based on LLM) |
| **A12** | Enhancing inquiry-based learning in human factors engineering with generative AI: A case study in industrial design education | Tseng et al. (2025) | Taiwan | Quantitative | Rubric | 54 university students | ChatGPT (Conversat. based on LLM) |
| **A13** | Evaluating the impact of assistive AI tools on learning outcomes and ethical considerations in programming education | Park et al. (2025) | Canada | Mixed | Survey | 34 university students | GitHub Copilot  (GenAI) |
| **A14** | Everyone talks everything with ChatGPT | Petrič (2024) | Slovenia | Quantitative | Scale | 340 university students | ChatGPT (Conversat. based on LLM) |
| **A15** | Examining generative AI user addiction from a C-A-C perspective | Zhou & Zhang (2024) | China | Quantitative | Scale | 529 adults | GenAI |
| **A16** | Exploring artificial intelligence (AI) Chatbot usage behaviors and their association with mental health outcomes in Chinese university students | Zhang et al. (2025) | China | Quantitative | Survey | 1004 university students (460 users) | Chatbot |
| **A17** | Exploring the relationship between AI literacy, AI trust, AI dependency, and 21st century skills in preservice mathematics teachers | Zhang et al. (2025) | China | Quantitative | Survey | 50 preservice teachers | GenAI |
| **A18** | Friend, mentor, lover: does chatbot engagement lead to psychological dependence? | Xie et al. (2023) | USA | Mixed | Survey and interviews | 123 (+ 14 interviews) users | Replika  Social chatbot |
| **A19** | From assistance to reliance: Development and validation of the large language model dependence scale | Li et al. (2025) | China | Quantitative | Survey | 421+ 1030 adults | LLM |
| **A20** | How do personal attributes shape AI dependency in Chinese higher education context? Insights from needs frustration perspective | Zhong et al. (2024) | China | Quantitative | Survey | 958 university students | ChatGPT (Conversat. based on LLM) |
| **A21** | How social anxiety leads to problematic use of conversational AI: The roles of loneliness, rumination, and mind perception | Hu et al. (2023) | China | Quantitative | Survey | 516 adults | Conversat. AI |
| **A22** | Influence of self-efficacy in the use of artificial intelligence (AI) and anxiety toward AI use on AI dependence among Peruvian university students | Morales-García et al. (2025) | Perù | Quantitative | Survey | 528 university students | Not specified |
| **A23** | It’s scary to use it, it’s scary to refuse it: The psychological dimensions of AI adoption—anxiety, motives, and dependency | Frenkenberg & Hochman (2025) | Israel | Quantitative | Survey | 242 adults | Conversat. AI |
| **A24** | Latent profile analysis of AI literacy and trust in mathematics teachers and their relations with AI dependency and 21st-century skills | Wijaya et al. (2024) | China | Quantitative | Survey | 469 math teachers | GenAI |
| **A25** | Metacognitions about generative AI use: psychometric and network analysis among Chinese college students | Xie et al. (2025) | China | Quantitative | Survey | 1229 college students | GenAI |
| **A26** | Modeling the influence of AI dependence to research productivity among STEM undergraduate students: case of a state university in the Philippines | Buniel et al. (2025) | Philippines | Quantitative | Survey | 834 university students | Not specified |
| **A27** | One is the loneliest number… Two can be as bad as one. The influence of AI Friendship Apps on users' well‐being and addiction | Marriott & Pitardi (2024) | UK | Mixed | Survey, posts, semi-structured interviews | 572 (+21 interviews) | Replika  social chatbot |
| **A28** | Parental and peer phubbing and college students’ gen AI dependency: the mediating roles of loneliness and self-efficacy and the moderating role of perception of gen AI | Liao et al. (2025) | China | Quantitative | Survey | 1928 university students | GenAI |
| **A29** | Problematic ChatGPT Use Scale: AI-human collaboration or unraveling the dark side of ChatGPT | Maral et al. (2025) | Turkey | Quantitative | Survey | 391 + 473 adults | ChatGPT (Conversat. based on LLM) |
| **A30** | Social chatbot: My friend in my distress | Ali et al. (2023) | China | Quantitative | Survey | 366 university students | Xiaoice  (social chatbot) |
| **A31** | Students’ AI Dependency in 3R’s: Questionnaire Construction and Validation | Capinding (2024) | Philippines | Mixed | Survey and interviews | 1635 university students (6 students and 12 teachers interviews) | AI powered tools for reading, writing and numeracy |
| **A32** | The development and validation of an artificial intelligence chatbot dependence scale | Zhang et al. (2025) | China | Quantitative | Survey | 233 adults | Chatbot |
| **A33** | The mediating role of academic stress, critical thinking and performance expectations in the influence of academic self-efficacy on AI dependence: Case study in college students | Acosta-Enriquez et al. (2025) | Perù | Quantitative | Survey | 676 university students | Not specified |
| **A34** | The paradox of self-efficacy and technological dependence: Unraveling generative AI's impact on university students' task completion | Zhang & Xu (2025) | China | Quantitative | single item | 200 university students | GenAI |
| **A35** | Transformative pedagogy in the digital age: Unraveling the impact of artificial Intelligence on higher education students | Capinding & Dumayas (2024) | Philippines | Quantitative | Survey | 194 university students | Not specified |
| **A36** | Who is hooked on AI? The role of the big five personality traits in compulsive ChatGPT use among Chinese students | Hu et al. (2025) | China | Quantitative | Survey | 247 university students | ChatGPT (Conversat. based on LLM) |
| **A37** | “incomplete without tech”: Emotional responses and the psychology of AI reliance | Biswas & Murray (2025) | UK | Mixed | Survey | 65 adults | Not specified |
